# Examining the relations between teacher level factors and the use of spelling instruction and assessment practices

**DOI:** 10.1007/s11881-026-00381-2

**Published:** 2026-07-23

**Authors:** Ramona T. Pittman, Heesun Chang, Kimberly Wright, Misty Stiles, Taylor Seymour, Emily Binks-Cantrell, R. Malatesha Joshi

**Affiliations:** 1 Department of Teaching, Learning & Culture, Texas A&M University, College Station, USA; 2 Department of Educational Psychology, Texas A&M University, College Station, USA

**Keywords:** Spelling, Spelling instruction, Spelling activities, Spelling assessment, Teacher knowledge

## Abstract

This study investigated elementary teachers’ spelling activity and assessment practices and examined how these practices relate to teachers’ spelling knowledge, self-efficacy in teaching spelling, and instructional philosophy. Survey responses were collected from 324 K to 5 teachers in a diverse urban district, including open-ended descriptions of spelling activities and scoring methods. Responses were analyzed and categorized into eight instructional activity types and five scoring approaches. Descriptive and multinomial logistic regression analyses revealed that teachers’ activity choices were more strongly associated with their self-efficacy and philosophy in teaching spelling than with their formal spelling knowledge. Teachers with stronger philosophical beliefs were more likely to report linguistically informed practices (e.g., Word Sorts), while those with higher self-efficacy tended to favor traditional spelling activities (e.g., worksheet-based tasks). Scoring practices showed a preference for partial-credit approaches, though many still used traditional right/wrong grading. Spelling knowledge showed a marginal association with nuanced scoring that goes beyond mere right-or-wrong evaluations, but all three traits (i.e., spelling knowledge, self-efficacy, and philosophy) did not significantly predict scoring methods. These findings suggest that teacher education should address not only spelling knowledge but also self-efficacy and philosophy to support evidence-based spelling practices.

Spelling is frequently overlooked in the larger context of literacy instruction (Joshi et al., 2008), yet it plays a central role in reading and writing development (Abbot et al., 2010; Graham & Santangelo, 2014). Research demonstrates that explicit spelling instruction strengthens students’ reading by reinforcing phoneme–grapheme correspondences, a process essential for decoding (Graham & Santangelo, 2014; Weiser & Mathes, 2011). When integrated with phonics, spelling improves fluency, enabling faster word recognition and ultimately supporting comprehension (Graham & Hebert, 2010; Moats, 2005/2006; Weiser & Mathes, 2011). In writing, spelling proficiency reduces the cognitive load of transcription, freeing students to focus on higher-level composition, which improves overall writing quality (Berninger et al., 2013; Graham et al., 2018).

Inappropriate spelling instruction, however, can negatively impact students’ literacy development and overall engagement with language (Bear et al., 2011; Moats, 2020; Graham et al., 2008). One of the most problematic approaches to spelling instruction is rote memorization, where students are expected to memorize long lists of words without understanding the underlying spelling patterns or rules (Joshi et al., 2008). This method focuses solely on recall, which leads to poor retention and limited ability to transfer these skills to new or unfamiliar words. For example, teachers often assign visually engaging activities, such as triangular spelling (i.e., students write a word in the shape of a triangle by adding one letter at a time), rainbow spelling (i.e., students use different colors to write each letter of a word), etc., which emphasize surface-level engagement with words rather than fostering a deep understanding of language. These methods (e.g., triangular and rainbow spelling) do not contribute meaningfully to students’ ability to understand the relationships between letters and sounds, which is critical for reading and writing development (Al Otaiba et al., 2010). Studies have shown that these methods and rote memorization are less effective in fostering long-term spelling proficiency compared to methods that engage students in understanding phonemic, morphemic, and orthographic patterns (Berninger et al., 2010; Ehri, 2000; Graham & Santangelo, 2014; National Research Council et al., 1998; Weiser & Mathes, 2011).

Furthermore, the importance of spelling instruction extends beyond school performance. Despite the prevalence of digital tools such as spellcheck and autocorrect, proficiency in spelling remains essential in academic and professional contexts. Spelling accuracy influences perceptions of competence and credibility, with errors often diminishing how students and adults are viewed in formal communication (Graham et al., 2014; Pan et al., 2021). As such, spelling instruction is not simply about word lists but about developing the knowledge that shapes literacy achievement and future opportunities.

In addition, assessment practices provide a critical link between spelling instruction and literacy outcomes (McNeill & Kirk, 2014). Principle-based or qualitative scoring reveals the linguistic knowledge that students bring to their spelling attempts (Masterson & Apel, 2010). For example, a child spelling *cat* as *kat* demonstrates orthographic understanding but needs targeted instruction on when to use the letters “c” (before a, o, u or a consonant; *cat*,* cop*,* cut*,* clean*) or “k” (before e, i, or y; *key*,* kite*,* sky*) to represent the initial /k/ in *cat*, whereas a spelling such as *xjb* for *cat* indicates foundational phonemic awareness gaps and the child’s inability to map the correct phonemes with its matching graphemes. These distinctions are vital for informing differentiated instruction. However, when teachers rely solely on rote memorization tasks or binary right or wrong scoring, they risk overlooking diagnostic insights and limiting opportunities for differentiated instruction (Schlagal, 2007; Weiser & Mathes, 2011).

Beyond assessment, it is important to consider how teachers make instructional decisions about spelling. While knowledge of phonological, morphological, etymological, and orthographic structures is necessary for research-based spelling instruction (Joshi et al., 2008; Pittman et al., 2014; Ehri, 2014), research suggests that knowledge alone does not fully explain teachers’ choices (McNeil & Kirk, 2014). Teachers’ practices are also shaped by their philosophical beliefs about spelling and their confidence in teaching it (Pittman et al., 2023). Self-efficacy theory (Bandura, 1977) highlights the importance of this confidence since individuals who believe they can perform a task are more likely to persist, adapt strategies, and succeed. In education, teacher self-efficacy has been linked to greater adoption of research-based practices and persistence when facing challenges (Tschannen-Moran & Hoy, 2001). In spelling specifically, teachers with higher self-efficacy may rely on familiar spelling routines (e.g., rote memorization of spelling lists), while those with strong beliefs in linguistic instruction may favor word study or phoneme–grapheme mapping. Thus, the spelling activities teachers assign are not incidental, as they can be shaped by their self-efficacy and philosophical beliefs.

Collectively, these factors highlight the need to examine teachers’ spelling practices in greater depth. Instructional activities and assessment methods are not only linked to students’ literacy outcomes but can also reflect a relationship to teacher knowledge, self-efficacy, and philosophy. This study therefore investigates the types of spelling activities and scoring methods reported by elementary teachers and examines how these practices relate to teacher-level factors. By doing so, it contributes to a clearer understanding of the ways in which knowledge, beliefs, and confidence shape spelling instruction.

Our research questions, therefore, focused on spelling activities and spelling scoring methods. Thus, our research questions were:

1. What types of spelling activities do teachers use, and how are these choices associated with their knowledge, self-efficacy, and instructional philosophy?
  - What spelling activities/exercises do teachers use in general?
  - How does the use of these activities vary by teachers’ spelling knowledge?
  - How does the use of these activities vary by teachers’ self-efficacy?
  - How does the use of these activities vary by teachers’ philosophy?
2. What scoring practices do teachers use when assessing spelling, and how are these practices related to their knowledge, self-efficacy, and philosophy?
  - What scoring practices are used in classrooms?
  - How do these practices vary by teachers’ spelling knowledge?
  - How do these practices vary by teachers’ self-efficacy?
  - How do these practices vary by teachers’ philosophy?

## Method

### Participants and context

This study draws on data collected from a previous investigation (Pittman et al., 2023), which received IRB approval and examined teachers’ spelling knowledge, self-efficacy, and philosophy of spelling instruction using a teacher survey. The sample consisted of 324 K–5 teachers from a large urban school district in the southern United States. This district serves over 21,000 students across 15 elementary schools and employs more than 1,400 teachers. The student population was demographically diverse: 79.6% Hispanic, 15% African American, 3.8% White, and less than 1% each Asian, Native American, and multiracial. Approximately 86.9% of students were economically disadvantaged, 37.2% were classified as English learners, and over 36% participated in bilingual education programs.

The school district did not use a dedicated spelling curriculum; rather, spelling instruction was embedded within a popular reading curriculum. The spelling words were not organized around spelling patterns or generalizations but were instead drawn from vocabulary words tied to textbook readings, which simultaneously served as spelling words. To better understand teachers’ knowledge and instructional practices within this context, data were collected during a district-wide professional development (PD) session. The participating teachers completed the survey during the in-person PD session, which was focused on phonics instruction. Sessions were held over three days, with teachers grouped by grade level. The PD lasted three hours, and the survey was administered at the beginning of each session using participants’ electronic devices. Teachers were instructed not to discuss their responses while completing the survey.

Among the teacher participants, 53% were Hispanic (*n* = 171), followed by 19% African American (*n* = 62), 18% White (*n* = 59), and smaller percentages of Asian (2%), Native American (1%), and two or more races (3%). An additional 4% (*n* = 14) chose not to disclose their race or ethnicity. Most teachers (74%) held a bachelor’s degree (*n* = 239), while 26% (*n* = 85) held a master’s degree. Teachers reported a range of primary teaching assignments: 20% taught first grade (*n* = 64), 16% second grade (*n* = 51), 14% third grade (*n* = 45), 15% fourth grade (*n* = 47), 12% fifth grade (*n* = 40), and 15% kindergarten (*n* = 50). An additional 8% (*n* = 27) indicated other roles, such as interventionists, special education teachers, or those with multi-grade assignments. Teaching experience ranged from first-year teachers to those with up to 39 years of experience (M = 9.84, SD = 7.62).

### Survey instrument

The instrument included three validated scales that measured teachers’ spelling knowledge, self-efficacy in teaching spelling, and philosophical beliefs about spelling instruction (Pittman et al., 2023, 2026). The spelling knowledge measure consists of 26 dichotomously scored items assessing teachers’ knowledge of English spelling patterns, including phonological, orthographic, and morphological principles. The self-efficacy measure contains nine Likert-scale items assessing teachers’ confidence in teaching spelling-related skills, whereas the philosophy measure includes eight Likert-scale items examining teachers’ beliefs regarding the importance of linguistic and literacy components involved in spelling instruction. Rasch analyses were conducted to evaluate the psychometric quality of the three measures. A dichotomous Rasch model was applied to the spelling knowledge items, whereas two Rating Scale Models were used for the self-efficacy and philosophy measures, respectively. All three measures showed high item reliability (spelling knowledge = 0.98, self-efficacy = 0.98, philosophy = 0.99), and the model-data fit statistics supported unidimensionality for each construct, with all mean infit and outfit values close to the expected value of 1.00 across the three scales (spelling knowledge: infit *M* = 1.00, *SD* = 0.05, outfit *M* = 1.00, *SD* = 0.07; self-efficacy: infit *M* = 0.99, *SD* = 0.31, outfit *M* = 1.00, *SD* = 0.32; philosophy: infit *M* = 0.99, *SD* = 0.25, outfit *M* = 1.00, *SD* = 0.27). The participants’ scores for the three measures were used as predictors in the present study. Higher scores indicated stronger knowledge, greater instructional confidence, and stronger beli spelling instruction. Descriptive statistics for the three measures are presented in Table 1. For more details about the Rasch analyses of the three measures, please see Pittman et al. (2023).

**Table 1:** Summary statistics of teachers’ spelling knowledge, self-efficacy, and philosophy measures (Pittman et al., 2023)

Measure	M	SD	Min	Max
Spelling Knowledge	-0.52	0.69	-2.20	2.31
Self-efficacy	0.75	1.39	-5.14	5.50
Philosophy	2.59	1.27	-0.21	6.01

In Pittman et al. (2023), the survey also included two open-ended questions: (1) “List and briefly explain the top two spelling activities/exercises you have students complete,” and (2) “If you answered that you ‘always, most of the time, about half the time, or sometimes’ use a scoring protocol/procedure/rubric other than scoring a word as ‘right’ or ‘wrong,’ please briefly describe what you use.”

The participants’ responses to the first item were reviewed by two graduate researchers. If a teacher listed more than one activity in a single response, these were separated and treated as distinct entries. After independently identifying initial themes, the coders collaborated to refine and define each category. With guidance from a field expert, they finalized eight categories: Word Sort, Repetition, Working with Words and Sounds, Games and Centers, Early Spellers/Inventive Spelling, Traditional Activity, Do Not or Have Not Taught Spelling, and Could Not Be Determined. For instance, activities involving phoneme-grapheme mapping or letter tiles were classified as Working with Words and Sounds, while practices like spelling tests, ABC order, or using a dictionary were categorized as Traditional Activities. Final coding was conducted independently by both researchers, and discrepancies were resolved in discussion. The intercoder reliability was 0.94 (Cohen’s Kappa), indicating strong agreement. Table 2 presents the finalized activity categories.

**Table 2:** Spelling activity variables and descriptions

Variable	Description	Example Responses
Word Sorts	Sorting words by common patterns	Words Their Way
Repetition	Repeatedly writing the word	Rainbow writing, copying words multiple times
Working With Words and Sounds	Instruction recognizing sounds, syllables, patterns in words	Elkonin boxes, letter tiles, highlighting targeted phonemes, word building, say it-build it-spell it
Games and Centers	Student-led activities	Tic-Tac-Toe, Bingo, Scrabble, Spelling with A Twist
Early Spellers/Inventive Spelling	Inventive spelling based on sounds	Create their own words, most kindergarten spelling is inventive
Traditional	Uses spelling words but emphasizes a different skill	ABC order, spelling test, definitions, use word in a sentence, homework practice
Do Not or Have Not Taught Spelling	Not teaching spelling	First year teacher, changed grade levels, I don’t teach spelling, never been in ELA, N/A
Could Not Be Determined	Vague or unrelated response	Spanish, gesture related to words

The type of spelling activities teachers used categorized into themes/variables

A similar process was followed for the second open-ended item regarding scoring practices. Although the question asked for alternatives to right or wrong grading, several teachers still described traditional methods. The researchers initially coded responses independently, then finalized five categories: Traditional/Regular Grading Scale, Partial Credit/Score Based on Principle, Correct Usage/Definition/Sentence, No Assessment, and Could Not Be Determined. Traditional responses referred to correct or incorrect marking, while partial credit methods reflected an awareness of phonological or orthographic approximations. Context-based scoring involves assessing spelling in sentence use or journal writing. The intercoder reliability for this coding task was perfect (Cohen’s Kappa = 1.00), confirming complete agreement between coders.

### Analytic approach

Descriptive statistics were used to summarize the types of spelling activities and scoring practices teachers reported. To examine how these practices varied by teacher characteristics, cross-tabulations were conducted comparing the frequency of activity and scoring types across high and low groups of spelling knowledge, self-efficacy, and instructional philosophy, based on participants’ Rasch-scaled scores of the three traits (Pittman et al., 2023). To further investigate whether teachers’ spelling knowledge, self-efficacy, and instructional philosophy predicted their instructional and scoring practices, multinomial logistic regression models were conducted for both spelling activities and scoring practices; all models used the participants’ Rasch scores for the three traits as predictors (Pittman et al., 2023).

## Results

Research Question 1: What types of spelling activities do teachers use, and how are these choices associated with their knowledge, self-efficacy, and instructional philosophy?

### Frequency of spelling activities

One hundred and sixty teachers (49% of the 324 teachers) responded to the survey question asking them to provide two descriptions of their spelling activities or exercises. Among them, 32 teachers provided only one activity, and 13 teachers indicated they did not have any teaching experience. A total of 262 spelling activities were reported. As shown in Table 3, the most commonly reported activities fell into the Traditional category (28%), which included practices such as placing words in alphabetical order, assigning spelling tests, writing definitions, or using spelling words in sentences. These activities reflect a more conventional approach to spelling instruction that emphasizes memorization and rote practice.

**Table 3:** Frequency distribution of spelling activities

Spelling Activity Type	Frequency (%)
Word sort	38 (15%)
Repetition	45 (17%)
Working with words and sounds	70 (27%)
Games and centers	27 (10%)
Early spellers/inventive spelling	3 (1%)
Traditional activity	73 (28%)
Could not be determined	6 (2%)
Total	262 activities reported
Do not or have not taught spelling	13
Number of teachers who indicated only one activity	32
No response given	164

161 teachers responded by providing 262 total activities

Closely following Traditional activities were those categorized as Working with Words and Sounds (27%). This group involved phonics-based strategies and activities designed to strengthen students’ understanding of phoneme-grapheme correspondences. Examples include highlighting targeted phonemes, using Elkonin boxes, or having students manipulate letter tiles to build words. These activities align more closely with structured literacy approaches and emphasize foundational decoding and encoding skills.

Repetition activities, such as rainbow writing or repeatedly copying spelling words, accounted for 17% of all responses. While these methods promote practice through visual or motor repetition, they may not explicitly reinforce the linguistic principles underlying correct spelling. Word Sorts, which constituted 15% of the activities, typically involved categorizing words based on spelling patterns or phonological rules. This activity (e.g., *Words Their Way*) encourages analytical thinking about word structures. Activities in the Games and Centers category made up 10% of the total responses and included examples, such as spelling tic-tac-toe, bingo, and word-based games implemented as part of literacy centers. These activities are often designed to make spelling practice more engaging but vary widely in their alignment with evidence-based practices.

Only 1% of activities were classified as Early Spellers/Inventive Spelling, which included phonetic approximations by younger students based on their current stage of language development. Additionally, 2% of responses were placed in the “Could Not Be Determined” category due to vagueness or ambiguity, and 13 teachers (not included in the total count of 262 activities) reported not teaching spelling at all. Overall, the data suggest a strong reliance on conventional and phonics-based methods, with fewer teachers employing approaches that integrate inventive or exploratory spelling strategies.

### Activity use by teacher knowledge, self-efficacy, and philosophy

Table 4 displays the distribution of spelling activity types across teachers grouped by high and low levels of spelling knowledge, self-efficacy, and philosophy. They were divided into two groups based on the average score of each measure: a higher group whose scores were above the average score of spelling knowledge, self-efficacy, and philosophy, respectively, vs. a lower group whose scores were below the average score of each measure. Both groups across all three traits were generally comparable in terms of background characteristics. Each group included teachers from across all elementary grade levels and other instructional roles. Racial and ethnic as well as their degree attainment distributions were similarly balanced, suggesting overall comparability between the groups.

**Table 4:** Activity distribution across teachers’ spelling knowledge, self-efficacy, and philosophy

Activity	Spelling Knowledge		Self-efficacy		Philosophy	
	Higher	Lower	Higher	Lower	Higher	Lower
WS	19% (*n* = 26)	10% (*n* = 12)	15% (*n* = 16)	14% (*n* = 22)	19% (*n* = 25)	10% (*n* = 13)
R	19% (*n* = 26)	16% (*n* = 19)	15% (*n* = 16)	19% (*n* = 29)	16% (*n* = 21)	19% (*n* = 24)
WWS	27% (*n* = 38)	27% (*n* = 32)	32% (*n* = 33)	24% (*n* = 37)	29% (*n* = 38)	26% (*n* = 32)
GC	10% (*n* = 14)	11% (*n* = 13)	10% (*n* = 10)	11% (*n* = 17)	11% (*n* = 14)	10% (*n* = 13)
ESIS	0% (*n* = 0)	3% (*n* = 3)	2% (*n* = 2)	1% (*n* = 1)	2% (*n* = 3)	0% (*n* = 0)
TA	25% (*n* = 35)	32% (*n* = 38)	26% (*n* = 27)	30% (*n* = 46)	23% (*n* = 30)	34% (*n* = 43)
Total	139	117	104	152	131	125

*WS* Word Sort, *R* Repetition, *WWS* Working with Words and Sounds, *GC* Games and Centers, *ESIS* Early Spellers/Inventive Spelling, and *TA* Traditional Activity

Teachers with higher spelling knowledge tended to use more Word Sort and Repetition activities, whereas those with lower spelling knowledge more frequently used Traditional methods. Teachers with higher self-efficacy were more likely to report Working with Words and Sounds activities, while those with lower self-efficacy reported more Traditional and Repetition activities. In terms of philosophy, teachers who strongly believed in the importance of teaching spelling were more inclined to use Word Sort and Working with Words and Sounds activities, whereas those with weaker beliefs more often reported using Repetition and Traditional activities. Across all three traits, lower trait levels were associated with more frequent use of Traditional methods, whereas Games and Centers and Inventive Spelling activities were relatively unaffected by teacher characteristics.

### Multinomial logistic regression results for activity practices

Two sets of multinomial logistic regression models were conducted (see Table 5): one for the first reported activity and another for the second. Because participants were asked to provide two activities without ranking them by frequency of use, preference, or importance, the first and second reported activities should not be interpreted as representing primary and secondary activities. Due to low frequency, Early Spellers/Inventive Spelling in the first set and Word Sort in the second were excluded from the models. The first model fit the data adequately (*Pearson χ²*(520) = 523.04, *p* = .454; *Deviance χ²*(520) = 410.74, *p* = 1.00), though it did not significantly improve the intercept-only model (*χ²*(12) = 15.45, *p* = .218). Despite this, meaningful patterns were observed in the comparisons between specific activity categories.

**Table 5:** Frequency of two sets of spelling activities/exercises

Activity	Set 1	Set 2
WS	33	5
R	21	24
WWS	36	34
GC	17	10
ESIS	3	0
TA	35	38
Total	145	111

*WS* Word Sort, *R* Repetition, *WWS* Working with Words and Sounds, *GC* Games and Centers, *ESIS* Early Spellers/Inventive Spelling, and *TA* Traditional Activity

Philosophy of teaching spelling and self-efficacy were more associated with teachers’ activity preferences than spelling knowledge, which did not significantly predict instructional choices. Teachers with stronger philosophical beliefs were significantly more likely to report using Word Sorts rather than Working with Words and Sounds (*B* = 0.423, *p* = .041, Exp(*B*) = 1.53, *95% CI* [1.02, 2.29]) and Traditional Activities (*B* = 0.502, *p* = .020, Exp(*B*) = 1.65, *95% CI* [1.08, 2.52]). A marginal effect was also observed in the comparison between Word Sorts and Games and Centers (*B* = 0.434, *p* = .093, Exp(*B*) = 1.54, *95% CI* [0.93, 2.56]), i.e., more likely for teachers with stronger beliefs in teaching spelling to prefer Word Sorts over Games/Centers.

Self-efficacy significantly predicted lower odds of using Word Sorts (*B* = -0.476, *p* = .027, Exp(*B*) = 0.62, *95% CI* [0.41, 0.95]) and Repetition (*B* = -0.535, *p* = .029, Exp(*B*) = 0.59, *95% CI* [0.36, 0.95]) relative to Traditional Activities, suggesting that teachers with greater self-efficacy in teaching spelling were more likely to favor familiar, rule-based routines (Traditional Activities) over Word Sorts or Repetition activities. In addition, a marginal effect was found when comparing Working with Words and Sounds to Repetition (*B* = 0.385, *p* = .103, Exp(*B*) = 1.47, *95% CI* [0.93, 2.34]), suggesting that more confident teachers may favor Working with Words and Sounds.

Notably, spelling knowledge did not significantly predict instructional activity choice in any model. However, a trend was observed in the contrast between Word Sorts and Traditional Activities (*B* = 0.710, *p* = .065, Exp(*B*) = 2.03, *95% CI* [0.96, 4.32]), indicating that teachers with higher spelling knowledge may be more inclined toward Word Sorts activities. All other pairwise comparisons in the model yielded non-significant results, indicating limited or no measurable differences in teachers’ choices of those activities across their spelling knowledge, self-efficacy, and philosophy levels.

Overall, these findings suggest more importance of instructional beliefs and teacher confidence, rather than spelling knowledge alone, in shaping classroom spelling practices. On the other hand, the second model (*Pearson χ²*(291) = 308.86, *p* = .226; *Deviance χ²*(291) = 257.41, *p* = .922) revealed no significant differences among the activity preferences.

Research Question 2: What scoring practices do teachers use when assessing spelling, and how are these practices related to their knowledge, self-efficacy, and philosophy?

### Frequency of scoring practices

Of the 324 teachers surveyed, 73 provided descriptions of their scoring practices. As shown in Table 6, the majority of these teachers (60%) reported using a Partial Credit/Score Based on Principle method. This category represented the largest proportion of responses, suggesting that a significant number of teachers attempt to evaluate spelling in a more nuanced way, rather than relying solely on dichotomous right/wrong judgments. The second most commonly reported method was the Traditional Regular Grading Scale, used by 21% of respondents, indicating that about one-fifth of the teachers continue to score spelling based on the total number of correct spellings without partial credit considerations.

**Table 6:** Frequency distribution of scoring practices

Scoring Practices	Frequency (%)
Traditional regular grading scale (correct/incorrect)	15 (21%)
Partial credit/score based on principle (inventive spelling, WTW)	44 (60%)
Correct usage/definition/sentence	5 (7%)
No assessment	2 (3%)
Could not be determined	7 (10%)
Total	73

Two hundred fifty-one teachers did not provide a response regarding their scoring practices

A much smaller group, 7% of teachers, reported using a Correct Usage/Definition/Sentence approach to scoring. This indicates that relatively few teachers consider spelling accuracy within the broader context of language use. Additionally, 3% of the teachers stated that they do not assess spelling at all, while 10% of the responses were placed in the Could Not Be Determined category due to vague or unclear descriptions (e.g., “sometimes,” “Yes”).

These results reflect a clear preference among responding teachers for scoring methods that allow for partial credit, though a sizable portion still relies on more traditional grading practices. Given the relatively low percentage of teachers who reported using contextual scoring methods or not assessing spelling at all, it appears that many teachers may approach spelling assessment as a discrete task rather than integrating it with broader language use.

### Scoring practices by teacher knowledge, self-efficacy, and philosophy

Table 7 presents the distribution of scoring methods by teacher characteristics. As in the case of spelling activities, the teachers were grouped based on the average score of each measure. Like the case of spelling activities, both groups were generally comparable in terms of their backgrounds (i.e., primary teaching grade, race/ethnicity, and degree attainment). These patterns suggest that differences in assessment practices are unlikely to be attributable to demographic or professional background differences.

**Table 7:** Scoring practice distribution across teachers’ spelling knowledge, self-efficacy, and philosophy

Scoring Practice	Spelling Knowledge		Self-Efficacy		Philosophy	
	Higher	Lower	Higher	Lower	Higher	Lower
TS	17% (*n* = 5)	29% (*n* = 10)	22% (*n* = 8)	26% (*n* = 7)	21% (*n* = 7)	27% (*n* = 8)
PS	79% (*n* = 23)	60% (*n* = 21)	68% (*n* = 25)	70% (*n* = 19)	71% (*n* = 24)	67% (*n* = 20)
CDS	3% (*n* = 1)	11% (*n* = 4)	11% (*n* = 4)	4% (*n* = 1)	9% (*n* = 3)	7% (*n* = 2)
Total	29	35	37	27	34	30

*TS* Traditional Regular Grading Scale (correct/incorrect), *PS* Partial Credit/Score Based on Principle, *CDS* Correct Usage/Definition/Sentence

Teachers with lower levels (below average) of spelling knowledge, self-efficacy, or philosophy were more likely to use the Traditional Regular Grading Scale. In contrast, those with higher levels (above average) of spelling knowledge and stronger instructional philosophy tended to use Partial Credit scoring approaches. Teachers with lower knowledge but higher self-efficacy were also more likely to report using scoring practices that emphasized the correct usage of words in context, such as in definitions or sentences.

### Multinomial logistic regression results for scoring practices

A multinomial logistic regression was conducted to examine whether teachers’ spelling knowledge, self-efficacy, and philosophy of spelling instruction predicted their self-reported scoring practices. The outcome variable included three categories: Traditional scoring (i.e., right/wrong marking), Partial credit based on principle (e.g., inventive spelling or phoneme-level analysis), and Correct usage or contextual scoring (e.g., sentence or definition-based assessment). The most frequently reported method was partial credit (68.8%), followed by traditional scoring (23.4%) and usage-based scoring (7.8%), as shown in Table 6. The model demonstrated adequate fit to the data based on goodness-of-fit statistics (*Pearson χ²*(116) = 117.77, *p* = .437; *Deviance χ²*(116) = 91.08, *p* = .958). However, the overall model did not significantly improve over the intercept-only model (*χ²*(6) = 8.15, *p* = .228).

To improve model stability, a follow-up binary logistic regression was conducted to compare only the two most frequently reported categories: traditional scoring and partial credit. A likelihood ratio chi-square test comparing the model to the intercept-only model yielded *χ²*(3) = 7.05, *p* = .070, indicating a marginal improvement in fit. Compared to the original three-category multinomial model (*χ²*(6) = 8.15, *p* = .228), the binary model demonstrated improved explanatory power. In this model, spelling knowledge again showed a marginal association (*B* = -1.034, *p* = .074, Exp(*B*) = 0.36, *95% CI* [0.11, 1.11]), indicating that teachers with greater spelling knowledge were less likely to use a right/wrong approach and more likely to adopt a partial-credit method.

All together, these results suggest that spelling knowledge may influence how teachers assess spelling, particularly in their likelihood to apply principle-based scoring rather than binary correctness. However, no significant effects were observed for self-efficacy or philosophy in shaping assessment practices.

## Discussion

Spelling supports literacy by linking decoding, fluency, and writing (Graham & Santangelo, 2014; Weiser & Mathes, 2011), while spelling assessment can guide instruction especially differentiated decoding and encoding instruction. This study, therefore, examined the spelling activities and assessment practices reported by K–12 teachers and investigated how the types of classroom activities and scoring practices they adopt vary as a function of their spelling knowledge, self-efficacy, and instructional philosophy.

The findings suggest that teachers’ beliefs and confidence may be more closely associated with reported spelling pedagogy than spelling knowledge alone, aligning with prior work highlighting the potential influence of teacher-level factors (Bandura, 1977; Tschannen-Moran & Hoy, 2001). Teachers most frequently reported using Working with Words and Sounds and Traditional spelling activities. Unfortunately, spelling instruction is often characterized by traditional routines such as memorization and rote practice (Joshi et al., 2008; Schlagal, 2007), and many teachers in this study reported using traditional activities such as writing sentences with spelling words and alphabetizing words. Repetition activities were also reported relatively frequently, whereas Inventive Spelling activities were reported least often. When teachers do not follow an evidence-based spelling curriculum or teach evidence-based spelling instruction by utilizing common the phonological morphological, etymological, and orthographic principles of spelling pertinent to spelling development, teachers resort to statements like English spelling is irregular and spelling words must be memorized (Joshi et al., 2008; Moats, 2009; Treiman, 1993). Additionally, they have students completing tasks such as copying each word five times each (Graham, 2000).

Descriptive analyses revealed several noteworthy patterns. Teachers with lower spelling knowledge, self-efficacy, or philosophy scores appeared more likely to report using Traditional spelling activities, whereas teachers with higher self-efficacy and philosophy scores appeared more likely to report using Working with Words and Sounds activities. Results from the multinomial logistic regression provided some evidence that teachers with stronger beliefs about spelling instruction were more likely to report incorporating Word Sort methods relative to Traditional activities and Working with Words and Sounds activities. Additionally, higher self-efficacy was associated with greater likelihood of reporting Traditional activities relative to Word Sort and Repetition activities. This pattern may reflect several plausible reasons: teachers with higher self-efficacy may demonstrate stronger commitment to familiar instructional routines developed through years of practice, may be responding to district-level curricular expectations that favor traditional approaches, or may draw confidence from prior training experiences that emphasized conventional literacy instruction. Self-efficacy beliefs are domain-specific and deeply shaped by mastery experiences, meaning that teachers who have built confidence through repeated use of traditional methods may be less inclined to adopt newer or less familiar approaches such as Word Sorts, regardless of their overall instructional competence (Bandura, 1997). This interpretation aligns with broader research on instructional change suggesting that confidence in one’s established range of practices can, somewhat counterintuitively, function as a barrier to the integration of more current evidence-based practices (e.g., Basckin et al., 2021; Guskey, 2002; Richardson & Placier, 2001). However, because the regression models demonstrated limited explanatory power, these findings should be viewed as preliminary and interpreted cautiously.

Importantly, causal relationships cannot be established from the present data. It is therefore not possible to determine whether teacher characteristics influence instructional choices or whether engagement in particular instructional practices are associated with differences in teachers’ confidence and beliefs about spelling instruction. Overall, the findings tentatively suggest that reported spelling activities may be more closely related to teachers’ self-efficacy and instructional philosophy than to their spelling knowledge, although additional research is needed to examine these relationships.

With only 73 teachers responding to the scoring practices open-ended question, the survey results indicated that many teachers relied on a “right” or “wrong” approach to scoring spelling. The lower response rate to this item compared to other survey questions could be for various reasons; for example, it was not required for the participants to respond to all the items to move to the next item, or perhaps participants did not know the answer. Among the reported scoring practices, however, Partial Credit/Score Based on Principle approaches (e.g., inventive spelling, Words Their Way) were reported most frequently, followed by Traditional Regular Grading Scale methods, while Correct Usage/Definition/Sentence approaches were reported least often. Descriptive statistics suggested that teachers with lower spelling knowledge, self-efficacy, or philosophy scores were more likely to report using Traditional Regular Grading Scale methods, whereas teachers with higher spelling knowledge and philosophy scores more frequently reported using Partial Credit/Scoring approaches. Because the statistical models provided only limited support for predictive relationships, these patterns should be interpreted as descriptive associations rather than evidence of causal or robust predictive effects. Future studies incorporating additional data sources, such as classroom observations, teacher interviews, and student spelling performance data, may help clarify these relationships and their implications for student learning.

### Limitations

This study has several limitations. First, the multinomial logistic regression models did not significantly improve upon the intercept-only model. A follow-up binary logistic regression comparing traditional and partial-credit scoring approaches showed marginal improvement in model fit. As a result, the observed associations between teacher characteristics (e.g., spelling knowledge, self-efficacy, and philosophy) and instructional or scoring practices should be interpreted cautiously. These findings should be considered preliminary and hypothesis-generating, and future studies with larger samples are needed to confirm whether teachers’ spelling knowledge reliably predicts their assessment practices. Additionally, not all teachers responded to the open-ended survey items, and the study relied exclusively on self-reported data. Without observational evidence or student-level outcomes, it remains unclear how reported practices align with actual classroom instruction and effectiveness. Finally, the cross-sectional design limits causal interpretations of the relationships between teacher characteristics and their instructional or assessment choices.

### Implications for research

Future research should move beyond self-reports to include direct observations of spelling instruction and, where possible, link teaching practices to student spelling performance. Such data would provide a more robust understanding of how teacher knowledge, self-efficacy, and instructional philosophy manifest in classroom practice. Furthermore, longitudinal and experimental studies are needed to determine whether increasing teachers’ knowledge or self-efficacy leads to more consistent use of evidence-based spelling activities and assessment methods. Future research could also explore how school and district-level support, such as curriculum mandates or professional learning communities, influence teachers’ ability to implement effective spelling instruction, given that many teachers rely on mandated curricula that may or may not systematically and explicitly address the phonological, morphological, etymological, and orthographic principles essential to spelling development.

### Implications for practice

First, descriptive patterns in the data suggested that teachers with lower levels of spelling knowledge were more likely to report relying on traditional practices, such as weekly spelling tests, sentence-writing with spelling words, or alphabetizing words. In contrast, teachers with higher levels of confidence and knowledge of spelling patterns and rules more frequently reported activities that emphasized sounds and letters. Given the limited explanatory power of the regression models, these findings should be interpreted cautiously. Nevertheless, they suggest that teacher preparation programs may benefit from providing stronger foundations in English language structure and evidence-based spelling instruction.

It should be noted, however, that research examining the spelling knowledge of teacher educators (Pittman et al., 2022) found that many teacher educators reported limited preparation in spelling and did not routinely include spelling instruction in literacy coursework. Consequently, educator preparation programs (EPPs) may need to ensure that teacher educators themselves possess the linguistic knowledge necessary to support preservice teachers’ development. Therefore, it is important that EPPs ensure that teacher educators are equipped with the necessary spelling knowledge to teach preservice teachers. However, this is not without its challenges, as state policy often dictates what educator preparation programs are required to teach, which may limit the depth of spelling instruction embedded in preservice teacher training. At the school and district level, mandates that emphasize the science of reading should extend beyond reading to explicitly include spelling instruction. Spelling is deeply interconnected with early literacy development, yet it remains significantly underrepresented in assessment and instructional practices, which highlights the urgent need for districts to ensure that the science of reading encompasses spelling with the same rigor and intentionality applied to reading instruction (Seymour et al., 2026).

Additionally, distinguishing between spelling instruction and spelling practice opportunities remains important. Given the close relationship among reading, writing, and spelling, continued efforts to support in-service teachers in implementing evidence-based spelling practices may be warranted.

## Conclusion

Spelling instruction may require attention not only to teacher knowledge but also to teachers’ beliefs, self-efficacy, and instructional decision-making. Although the findings should be interpreted cautiously given the limited explanatory power of the statistical models, the results suggest that teachers’ instructional beliefs and self-efficacy may be associated with reported spelling activities and scoring practices. These associations appeared stronger than those observed for spelling knowledge alone, but additional research is needed to determine the robustness and direction of these relationships.

By offering a typology of spelling activities and assessment practices grounded in teachers’ own descriptions, this study contributes to the literature on an often-overlooked component of literacy instruction and provides a framework for future investigation. Collectively, the findings suggest that teacher preparation and professional development may benefit from integrating linguistic knowledge with opportunities to support teacher confidence and reflection on instructional decision-making. Future research is needed to determine whether such efforts are associated with greater implementation of evidence-based spelling instruction and improved student literacy outcomes.

## Data Availability

No datasets were generated or analysed during the current study.
